# Enhanced Electrochemical Performances of Mn_3_O_4_/Heteroatom-Doped Reduced Graphene Oxide Aerogels as an Anode for Sodium-Ion Batteries

**DOI:** 10.3390/nano12203569

**Published:** 2022-10-12

**Authors:** Nor Fazila Mahamad Yusoff, Nurul Hayati Idris, Muhamad Faiz Md Din, Siti Rohana Majid, Noor Aniza Harun

**Affiliations:** 1 Energy Storage Research Group, Faculty of Ocean Engineering Technology and Informatics, Universiti Malaysia Terengganu, Kuala Nerus 21300, Terengganu, Malaysia; 2 Department of Electrical and Electronic Engineering, Faculty of Engineering, National Defence University of Malaysia, Kem Sungai Besi, Kuala Lumpur 57000, Malaysia; 3 Center for Ionics University of Malaya, Department of Physics, Faculty of Science, University of Malaya, Kuala Lumpur 50603, Malaysia; 4 Advance Nano Materials (ANOMA) Research Group, Faculty of Science and Marine Environment, Universiti Malaysia Terengganu, Kuala Nerus 21300, Terengganu, Malaysia

**Keywords:** sodium-ion batteries, Mn_3_O_4_ nanoparticles, N-rGO aerogel, N,S-rGO aerogel, electrochemical performances

## Abstract

Owing to their high theoretical capacity, transition-metal oxides have received a considerable amount of attention as potential anode materials in sodium-ion (Na-ion) batteries. Among them, Mn_3_O_4_ has gained interest due to the low cost of raw materials and the environmental compatibility. However, during the insertion/de-insertion process, Mn_3_O_4_ suffers from particle aggregation, poor conductivity, and low-rate capability, which, in turn, limits its practical application. To overcome these obstacles, we have successfully prepared Mn_3_O_4_ nanoparticles distributed on the nitrogen (N)-doped and nitrogen, sulphur (N,S)-doped reduced graphene oxide (rGO) aerogels, respectively. The highly crystalline Mn_3_O_4_ nanoparticles, with an average size of 15–20 nm, are homogeneously dispersed on both sides of the N-rGO and N,S-rGO aerogels. The results indicate that the N-rGO and N,S-rGO aerogels could provide an efficient ion transport channel for electrolyte ion stability in the Mn_3_O_4_ electrode. The Mn_3_O_4_/N- and Mn_3_O_4_/N,S-doped rGO aerogels exhibit outstanding electrochemical performances, with a reversible specific capacity of 374 and 281 mAh g^−1^, respectively, after 100 cycles, with Coulombic efficiency of almost 99%. The interconnected structure of heteroatom-doped rGO with Mn_3_O_4_ nanoparticles is believed to facilitate fast ion diffusion and electron transfer by lowering the energy barrier, which favours the complete utilisation of the active material and improvement of the structure’s stability.

## 1. Introduction

Because of the rising demand for lithium-ion (Li-ion) batteries, the scarcity of lithium sources, and the expected steep rise in lithium prices, there is an urgent need for innovative and low-cost battery systems [1]. The most researched new battery technologies use the same insertion and extraction chemistry as Li-ion batteries, such as potassium-ion (K-ion) and sodium-ion (Na-ion) batteries, despite the fact that the electrode materials needed to be reconfigured [2]. In large-scale energy storage, Na-ion batteries have gained considerable interest owing to the availability and natural resources of sodium [3,4,5,6,7,8]; moreover, they appear to be a better alternative to Li-ion batteries. Unfortunately, because of the large radius (1.02 Å), high atomic mass (23 g mol^−1^), and low redox potentials (2.71 V vs. SHE) for Na-ion, most examined electrodes, especially the anode, are not ideal hosts for Na-ion insertion [9,10,11,12]. In addition, because Na has a higher chemical activity than Li, the production, transportation, and application of Na-ion batteries may be more difficult [13,14]. Furthermore, severe volume changes caused by repeated cycling of the anode materials contribute to the failure of the battery by causing cracks and loss of electrical contact. To improve battery performances, nanoscale materials are frequently used owing to their ease of stress release and high resistance to structural defect formation [15,16].

To date, various materials have been explored, and transition-metal oxides, such as MnO*_x_* [17,18], FeO*_x_* [19,20], and Co_3_O_4_ [21], have drawn increasing attention owing to their high theoretical capacities. Notably, previous studies have demonstrated that Mn and Mn-based compounds can alloy with Na and perform well as Na-ion battery anodes [15,22,23]. Mn_3_O_4_ is one of the promising anode materials and has a high theoretical capacity (937 mAh g^−1^) because of its conversion reaction mechanism, natural availability, low cost, and environmental friendliness [24]. So far, the Na-ion storage behaviour in Mn_3_O_4_ nanostructures has been investigated [25] and possesses similar problems to other transition-metal oxides. The volume changes during the repeated insertion/de-insertion process of Na-ions lead to the substantial aggregation of particles and may affect the mechanical stability of the electrode materials [26,27]. Jiang et al. [28] were the first to report the Na-ion storage performances of Mn_3_O_4_ thin film synthesised via electrostatic-spray deposition, which exhibits poor cycling performances. The capacity deterioration during cycling is explained by the formation of a porous reticular structure in the grids. To ensure the large capacity of Mn_3_O_4_ electrode accompanied by high reversibility [29], the internal structure of Mn_3_O_4_ needs to be modified to compensate for the volume changes by adopting various strategies, such as synthesise multifunctional nanostructures [25] and nanocomposites [30], and by hybridising with carbon-based materials, such as mesoporous carbon [31], graphene [32], reduced graphene oxide (rGO) [33], and carbon nanofibres [34]. Because of the unique layered structure, high specific area, superior conductivity, and excellent electrical properties, graphene-based matrices or their derivatives (e.g., rGO) have been used to improve the charge transfer and reduce the agglomeration of transition-metal oxide electrodes [35]. Such a combination could be an effective way to improve the electrochemical performances of the batteries by shortening the Na-ion diffusion pathway, improving electroactivity, and relieving volume variation [29,36]. Wang et al. [25] reported that encapsulating Mn_3_O_4_ nanotubes in porous graphene sheets can improve the structural integrity and electrical conductivity of the electrodes, with a satisfactory discharge capacity and cyclability of up to 55 cycles.

Nonetheless, aggregation or restacking between rGO layers will drastically reduce the active sites and influence the Na-ion transfer rate, which results in low reversible capacity [37]. Doping rGO with heteroatoms, such as nitrogen (N) [38,39] and sulphur (S) [40], could enhance the electronic conductivity and ameliorate the physicochemical functions of the rGO. It has been demonstrated that N-doping can significantly increase the electronic conductivity and Na-ion storage capacity of the electrode because of the introduction of N-doped defects and functionalised groups [41,42,43,44]. On the other hand, S-doping could increase the interlayer distance to promote the insertion/de-insertion of Na-ions owing to their larger covalent radius (102 pm) than carbon (77 pm) [43,45]. In addition, codoping with multiple heteroatoms can enhance the performance of rGO through synergistic interactions between heteroatoms [46]. However, the content of S in the doping materials could also be hindered by the incorporation of the graphene network because of the oversized atomic radius of S (180 pm) [47].

In this work, the Mn_3_O_4_/nitrogen (N)- and Mn_3_O_4_/nitrogen, sulphur (N,S)-rGO aerogels were prepared via the hydrothermal method using NH_3_ and *L*-cysteine as a source of nitrogen and nitrogen, sulphur elements, respectively. Upon treatment of graphene oxide (GO) with NH_3_ and *L*-cysteine, GO was not only reduced to graphene but also simultaneously doped with N and N,S-atoms. The Mn_3_O_4_/N- and Mn_3_O_4_/N,S-rGO aerogels have a promising reversible discharge capacity of 370 and 281 mAh g^−1^, respectively, up to 100 cycles at a current density of 0.1 A g^−1^. These nanocomposites also possess excellent cycling stability and better rate capability. In fact, when compared with other methods, the current method has the advantages of simplicity, friendliness, reliability, and cost-effectiveness. The promising capacity and cyclability demonstrated by the Mn_3_O_4_/heteroatom-doped rGO aerogel in our work add to the body of knowledge available to other researchers attempting to forecast the prospects of these composites for the development of Na-ion batteries.

## 2. Materials and Methods

### 2.1. Synthesis of the Mn_3_O_4_/N- and Mn_3_O_4_/N,S-rGO Aerogels

GO was obtained using a typical Hummers method [48]. Mn_3_O_4_ was synthesised according to the previous work [49]. For the synthesis of the Mn_3_O_4_/N-rGO aerogel, 90 mg GO and 63 mg Mn_3_O_4_ were ultrasonically dispersed in 18 mL deionised (DI) water for 1 h, and then 4 mL ammonia (NH_3_, Sigma-Aldrich, St. Louis, MO, USA) was slowly added into the mixture. The mixture was transferred into a Teflon-lined (125 mL) stainless-steel autoclave and heated at 180 °C for 12 h. Finally, the black hydrogel suspensions were freeze-dried to collect the Mn_3_O_4_/N-rGO aerogel. The same procedure was performed to prepare the N,S-rGO aerogel; however, NH_3_ was replaced with *L*-cysteine (Sigma-Aldrich, St. Louis, MO, USA) as a nitrogen/sulphur source and denoted as Mn_3_O_4_/N,S-rGO aerogel.

### 2.2. Physical Characterisation

The structural phases of the obtained samples were determined via X-ray diffraction (XRD; Rigaku Miniflex II, Tokyo, Japan). The amount of Mn_3_O_4_ in the heteroatom-doped rGO aerogel was confirmed using a thermogravimetric analyser (TGA, Perkin Elmer, Waltham, MA, USA) within the temperature range of 30 °C–800 °C at a heating rate of 10 °C min^−1^ in air. The morphology of these samples was observed via scanning electron microscopy (SEM; JOEL, Akishima, Tokyo, Japan) (JSM-6360L) and transmission electron microscopy (TEM; TECNAI G2 20 S-TWIN FEI, FEI Company, Lincoln, NE, USA). Fourier transform infrared spectroscopy (FTIR) was recorded on IR Tracer-100, Shimadzu, Kyoto, Japan. Raman spectra were collected via Raman spectroscopy (Renishaw, Gloucestershire, UK (532 nm radiation)) extended with 0.1% power laser measurement. Surface composition analysis was further conducted via X-ray photoelectron spectroscopy (XPS, Axis Ultra DLD XPS, Kratos, Manchester, UK).

### 2.3. Electrochemical Measurements

The active materials, carbon black (Sigma-Aldrich, >99.5%, St. Louis, MO, United States) and polyvinylidene fluoride (PVDF, Sigma-Aldrich, St. Louis, MO, United States) (weight ratio, 75:20:5), were dissolved in *N*-methyl-2-pyrrolidone (NMP). The slurry was then coated onto a copper (Cu) foil and dried at 100 °C overnight. CR2032 coin-type cells were fabricated in an argon-filled glovebox (Unilab, MBRAUN, Garching, Germany, H_2_O, O_2_ < 0.1 ppm) using sodium metal (Sigma-Aldrich, 99.9% trace metal base, St. Louis, MO, United States), glass fibre (GF/D Whatman) separator, and electrolyte (1 M NaClO_4_ (98%, Sigma-Aldrich) in propylene carbonate (99.7%, Sigma-Aldrich, St. Louis, MO, United States) with the addition of 5 wt.% fluoroethylene carbonate (99%, Sigma-Aldrich, St. Louis, MO, United States). The electrochemical performances of the nanocomposites were studied using a Neware battery analyser. Cyclic voltammetry (CV) was conducted using an electrochemical workstation (CHI 700E).

## 3. Results and Discussion

**Scheme 1:**
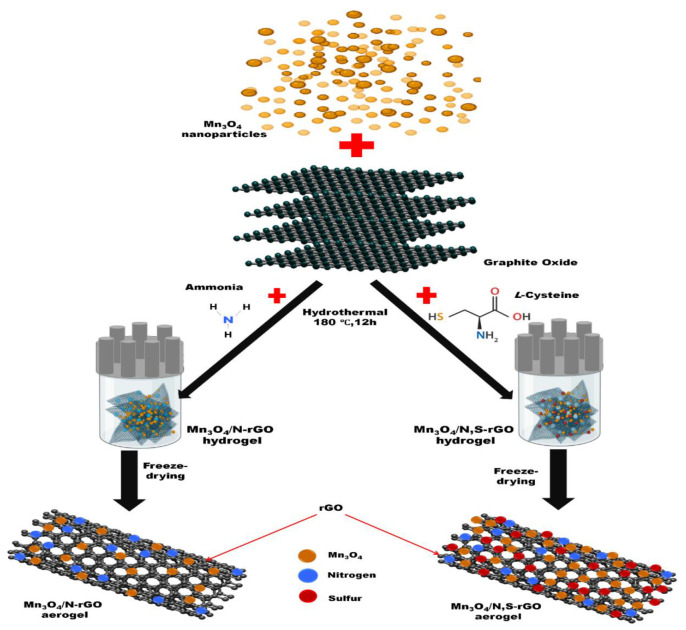
Schematic representation of the synthesis procedure of the Mn_3_O_4_/heteroatom-doped rGO aerogel.

Scheme 1 presents the synthesis of the Mn_3_O_4_/heteroatom-doped rGO aerogel using GO through hydrothermal, followed by freeze-drying. Upon heating, GO was converted to rGO, and simultaneously, through the π–π interactions, hydrogen bonding, coordination, and electrostatic interactions, the GO layers could self-assemble into three-dimensional (3D) networks. Concurrently, the presence of NH_3_ and *L*-cysteine introduced the doping of N-atom and N,S-atoms on the rGO layer, respectively. Strong cross-links, which are the building blocks of the 3D rGO network, were produced as a result of this process and acted as an effective conductive network for ion and electron transportation [30].

**Figure 1:**
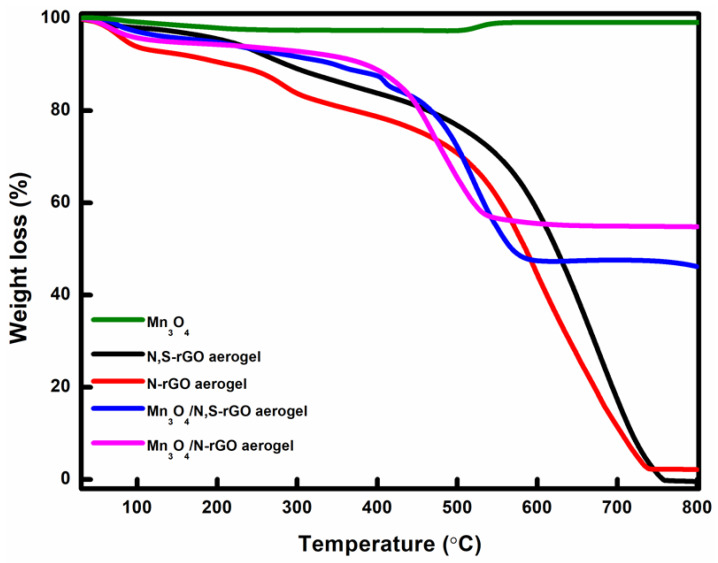
TGA curves of Mn_3_O_4_, N-rGO aerogel, N,S-rGO aerogel, Mn_3_O_4_/N-rGO aerogel, and Mn_3_O_4_/N,S-rGO aerogel at a heating rate of 10 °C min^−1^ in air.

The thermal stability (in air) of all samples was determined via TGA. The TGA curve (Figure 1) shows two weight loss stages for all samples, except for pristine Mn_3_O_4_, and the evaporation of physically and chemically adsorbed water was adequately attributed to the weight loss at temperatures above 100 °C. Between 150 °C and 600 °C, the N-rGO and N,S-rGO aerogels demonstrated the decomposition and disintegration of nitrogen and sulphur-containing functional groups, followed by decarboxylation and elimination of hydroxyl functionalities, respectively [50]. For a temperature less than 500 °C in air, rGO is often entirely burnt to CO_2_ [51]. For the Mn_3_O_4_ nanoparticles, no weight loss was observed in the temperature range of 100 °C to 500 °C. As the temperature approached 540 °C, the weight began to increase, which could be attributed to the transformation of Mn_3_O_4_ to Mn_2_O_3_ [30,33]. Therefore, the amounts of Mn_3_O_4_ in the Mn_3_O_4_/N-rGO and Mn_3_O_4_/N,S-rGO aerogels were estimated to be 63 and 60 wt.%, respectively.

**Figure 2:**
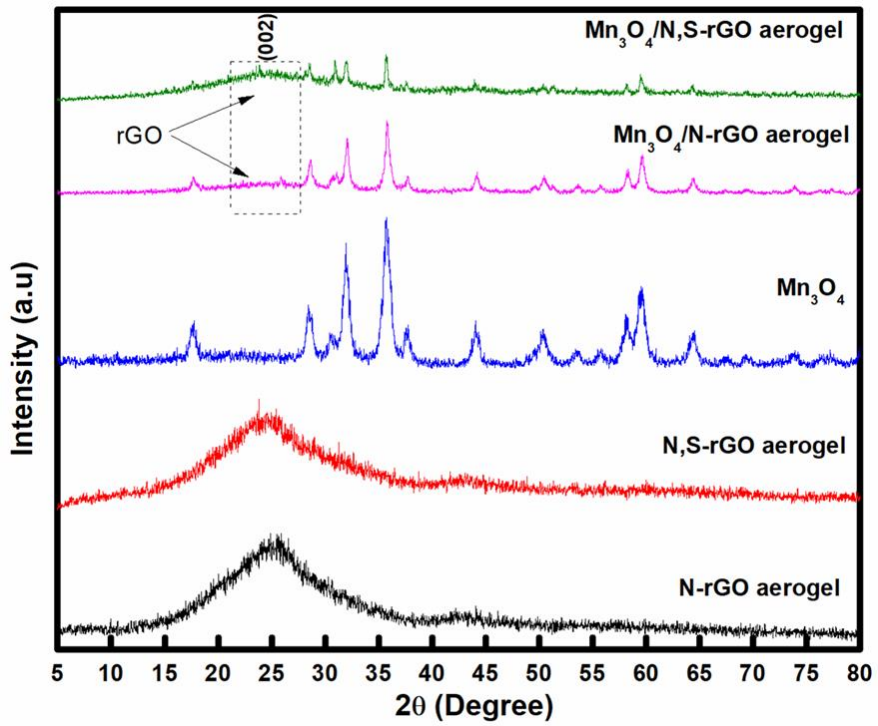
XRD patterns of Mn_3_O_4_, N-rGO aerogel, N,S-rGO aerogel, Mn_3_O_4_/N-rGO aerogel, and Mn_3_O_4_/N,S-rGO aerogel.

The phase purity and structure of the synthesised N-rGO aerogel, N,S-rGO aerogel, pure Mn_3_O_4_, Mn_3_O_4_/N-rGO aerogel, and Mn_3_O_4_/N,S-rGO aerogel were analysed via XRD. The disordered configuration of loosely packed graphene sheets of the N-rGO and N,S-rGO aerogels was disclosed by the enormous broad peak at approximately 24°–25°, corresponding to the graphite (002) plane (Figure 2) [52,53]. Because of the relatively low diffraction intensity of the N-rGO and N,S-rGO aerogels compared with Mn_3_O_4_ prominent peaks, the diffraction peaks of these aerogels were less evident in the XRD patterns of the Mn_3_O_4_/N-rGO and Mn_3_O_4_/N,S-rGO aerogels [54]. Both Mn_3_O_4_/N-rGO and Mn_3_O_4_/N,S-rGO aerogels corresponded to the planes of tetragonal crystallinity in Mn_3_O_4_ (JCPDS card no 240734), hence indicating the presence of pure Mn_3_O_4_ without any noticeable impurities. The crystallite size of Mn_3_O_4_/N-rGO and Mn_3_O_4_/N,S-rGO aerogels were 12.7 and 17.4 nm, respectively, calculated using Scherrer’s Equation. These results supported the formation and dispersion of Mn_3_O_4_ nanoparticles on the network surface of the N-rGO and N,S-rGO aerogels.

**Figure 3:**
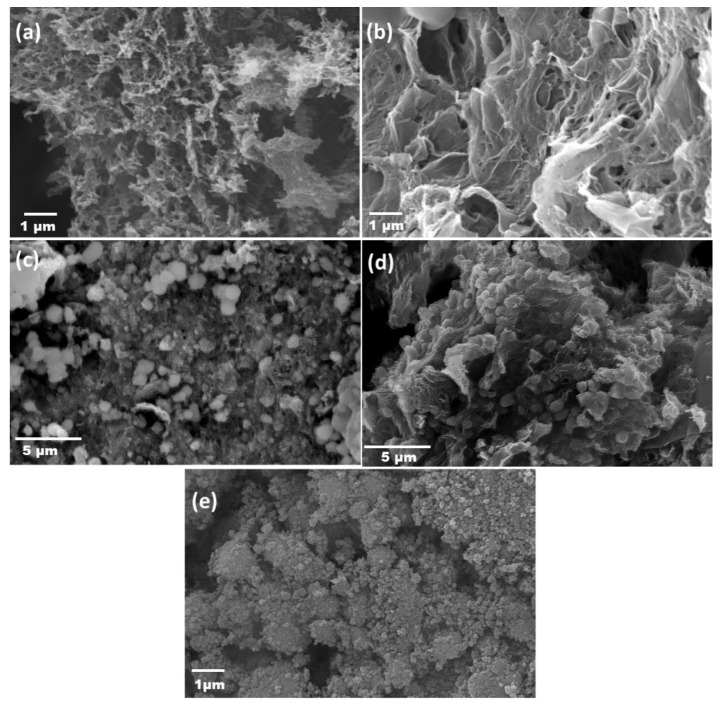
SEM images of (**a**) N-rGO aerogel, (**b**) N,S-rGO aerogel, (**c**) Mn_3_O_4_/N-rGO aerogel, (**d**) Mn_3_O_4_/N,S-rGO aerogel, and (**e**) pristine Mn_3_O_4_.

Figure 3 presents the SEM images for all samples. The N-rGO (Figure 3a) and N,S-rGO (Figure 3b) aerogel samples exhibited a typical well-defined and interconnected 3D network structure of rGO aerogel with a pore structure smaller than 1 µm. Such structures could provide an open channel for the access of electrolytes and minimise volume changes during the charge and discharge processes [55]. It is clearly demonstrated that Mn_3_O_4_ agglomerated in a size of 0.5–1.1 µm, which is an aggregate of individual Mn_3_O_4_ nanoparticles, anchored uniformly on the porous N-rGO (Figure 3c) and N,S-rGO (Figure 3d) aerogel layers. This suggests an effective assembly between the Mn_3_O_4_ nanoparticles and rGO aerogel sheets during the hydrothermal treatment. Moreover, pristine Mn_3_O_4_ nanoparticles with a particle size of 0.5–1.0 µm are beneficial in that they provide more active sites for the electrochemical reaction [56,57]. Therefore, the synergistic effect between the small-sized Mn_3_O_4_ and heteroatom-doped rGO aerogel could have a tremendous effect on the electrochemical properties, especially the cyclability and rate capability of the batteries.

**Figure 4:**
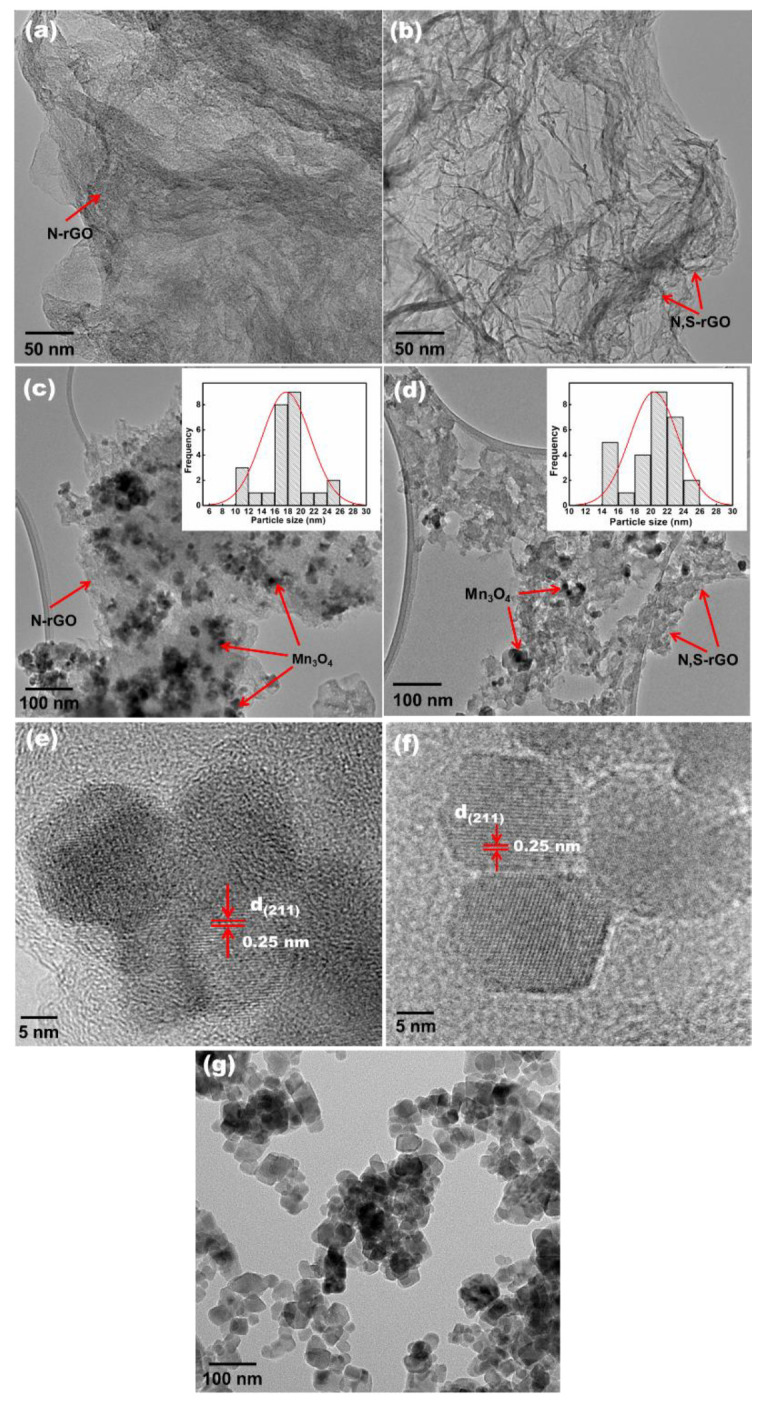
TEM images of (**a**) N-rGO aerogel, (**b**) N,S-rGO aerogel, (**c**) Mn_3_O_4_/N-rGO aerogel (inset: particle-size distribution), (**d**) Mn_3_O_4_/N,S-rGO aerogel (inset: particle-size distribution), and (**g**) Mn_3_O_4_ nanoparticles and HRTEM images of (**e**) Mn_3_O_4_/N-rGO aerogel and (**f**) Mn_3_O_4_/N,S-rGO aerogel.

Further characterisation of the morphology and structure of the Mn_3_O_4_/N-rGO and Mn_3_O_4_/N,S-rGO aerogels was carried out using high-resolution transmission electron microscopy (HRTEM). A typical crumpled structure of rGO and interconnected and cross-linked random rGO layers construct a 3D framework with open-pore structures (Figure 4a,b). The heteroatom-doped rGO aerogels exhibited a thin lamellar structure with distinct edges, overlaps, and curve profiles. Furthermore, the N-rGO and N,S-rGO aerogels had more wrinkled surfaces because of structural defects caused by heteroatom doping. Figure 4g shows the TEM image of Mn_3_O_4_. From Figure 4c,d, it can be seen that the Mn_3_O_4_ nanoparticles are uniformly dispersed over the surface of the rGO layers, with 15–20 nm average diameters of Mn_3_O_4_ nanoparticles (measured from the distribution histogram (inset)). The HRTEM images (Figure 4e,f) show that each Mn_3_O_4_ nanoparticle has a distinct lattice fringe, confirming the crystalline nature of Mn_3_O_4_ in the heteroatom-doped rGO aerogel. The HRTEM images indicate that the distinct interlayer *d*-spacing is 0.25 nm, which corresponds to the (211) plane of the tetragonal phase of the Mn_3_O_4_ nanoparticles. These results suggested that the Mn_3_O_4_ nanoparticles have a strong connection and network with the heteroatom-doped rGO aerogels, which is in accordance with the SEM images.

**Figure 5:**
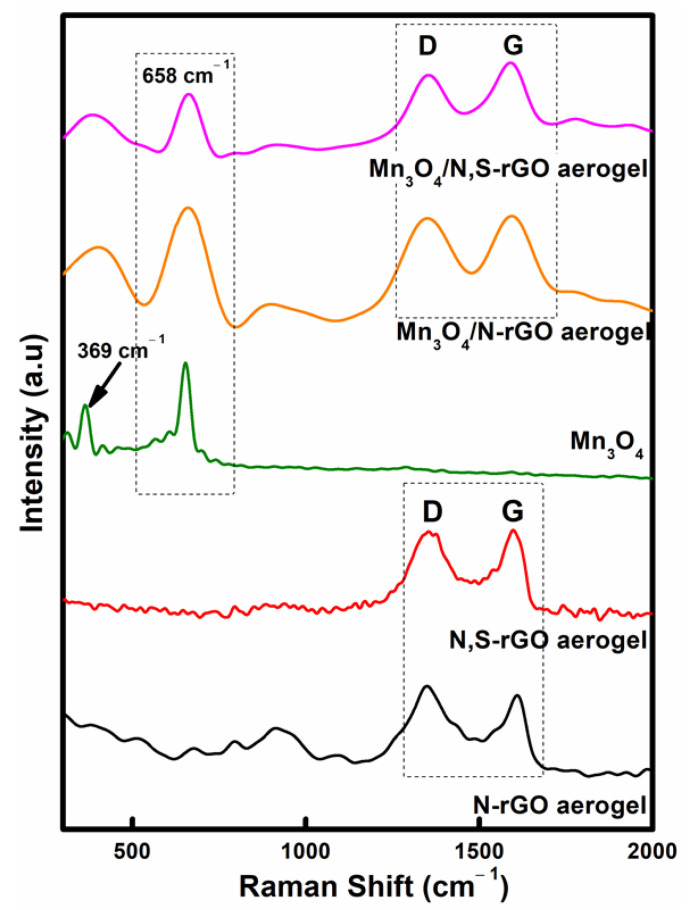
Raman spectra of N-rGO aerogel, N,S-rGO aerogel, Mn_3_O_4_ nanoparticles, Mn_3_O_4_/N-rGO aerogel, and Mn_3_O_4_/N,S-rGO aerogel.

The disordered degree of the heteroatom-doped rGO aerogels was characterised via Raman spectroscopy and is presented in Figure 5. Typical broad peaks corresponding to the D and G bands at 1359 and 1600 cm^−1^, respectively, were observed in the heteroatom-doped rGO aerogels. The G band reflected the radial C-C stretching of ordered sp^2^ -linked carbon atoms, whereas the D band indicated the defects or irregularities on the graphene edges [58,59]. Furthermore, the intensity ratio of the D and G bands (*I*_D_/*I*_G_) for the N-rGO aerogel was 0.95, whereas it was 0.98 for the N,S-rGO aerogel. After the nitrogen and sulphur doping, defects were introduced to the rGO aerogel layers [60,61], where the *I*_D_/*I*_G_ ratios of the Mn_3_O_4_/N-rGO and Mn_3_O_4_/N,S-rGO aerogels increased to 0.98 and 1.00, respectively. Two peaks at 658 and 369 cm^−^^1^ were observed in the Mn_3_O_4_/N-rGO and Mn_3_O_4_/N,S rGO aerogels, respectively [62,63]. These strong peaks corresponded to the Mn-O breathing vibration of Mn^2+^ ions and thus demonstrated that Mn_3_O_4_ is successfully attached to the rGO layer [64,65]. Additionally, the peaks at 2450 cm^−1^ were associated with the second-order two-phonon mode 2D band. It is worth noting that the Raman spectrum associated with Mn_3_O_4_ in the Mn_3_O_4_/N-rGO and Mn_3_O_4_/N,S-rGO aerogels was shifted to a low wavenumber in comparison with the pristine Mn_3_O_4_, indicating the electronic coupling between Mn_3_O_4_ and heteroatom rGO aerogel.

**Figure 6:**
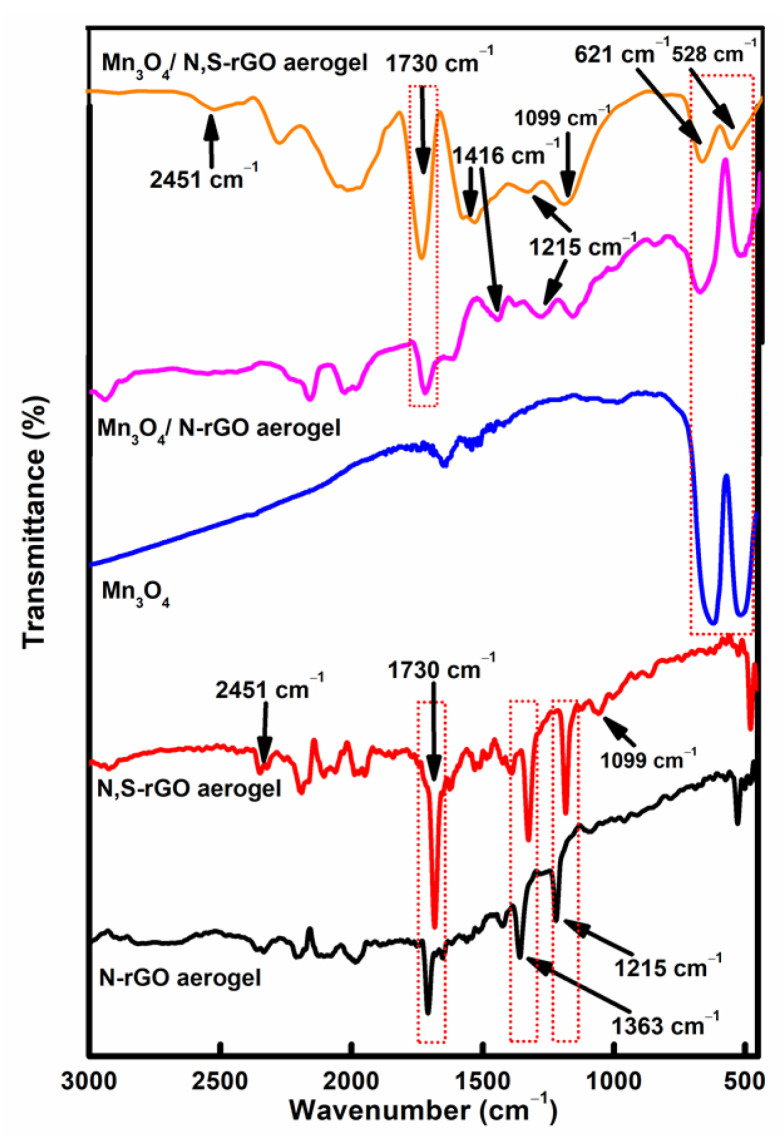
FTIR spectra of N-rGO aerogel, N,S-rGO aerogel, Mn_3_O_4_ nanoparticles, Mn_3_O_4_/N-rGO aerogel, and Mn_3_O_4_/N,S-rGO aerogel.

Figure 6 presents the FTIR spectra of the Mn_3_O_4_/N-rGO and Mn_3_O_4_/N,S-rGO aerogels to further support the presence of heteroatom in the rGO aerogel, as well as the Mn_3_O_4_ nanoparticles in the nanocomposites. The peak positioned at 1730, 1363, and 1215 cm^−1^ corresponded to the stretching vibration C=O of carboxylic groups, O-H deformation, and C-O stretching vibration from epoxy groups, respectively, indicating that GO was successfully converted into rGO [66,67]. The peak located at 1099 and 2451 cm^−1^ associated with the absorption band of C=S and S-H stretching vibration, respectively, was observed in the N,S-rGO and Mn_3_O_4_/N,S-rGO aerogels and, thus, confirmed the presence of S atom on the surface of N,S-rGO aerogel samples [68]. In addition, the C-N stretching vibration band located at 1416 cm^−1^ could be assigned to the characteristic band of nitrogen doping. For the Mn_3_O_4_ nanoparticles, the strong peaks at 528 and 621 cm^−1^ were attributed to the Mn-O stretching of the tetrahedral and octahedral sites in Mn_3_O_4_ [30]. As a result, Mn-O, C-N, C=S, and N-H linkages confirmed that Mn_3_O_4_ nanoparticles were successfully integrated into the heteroatom rGO layers.

**Figure 7:**
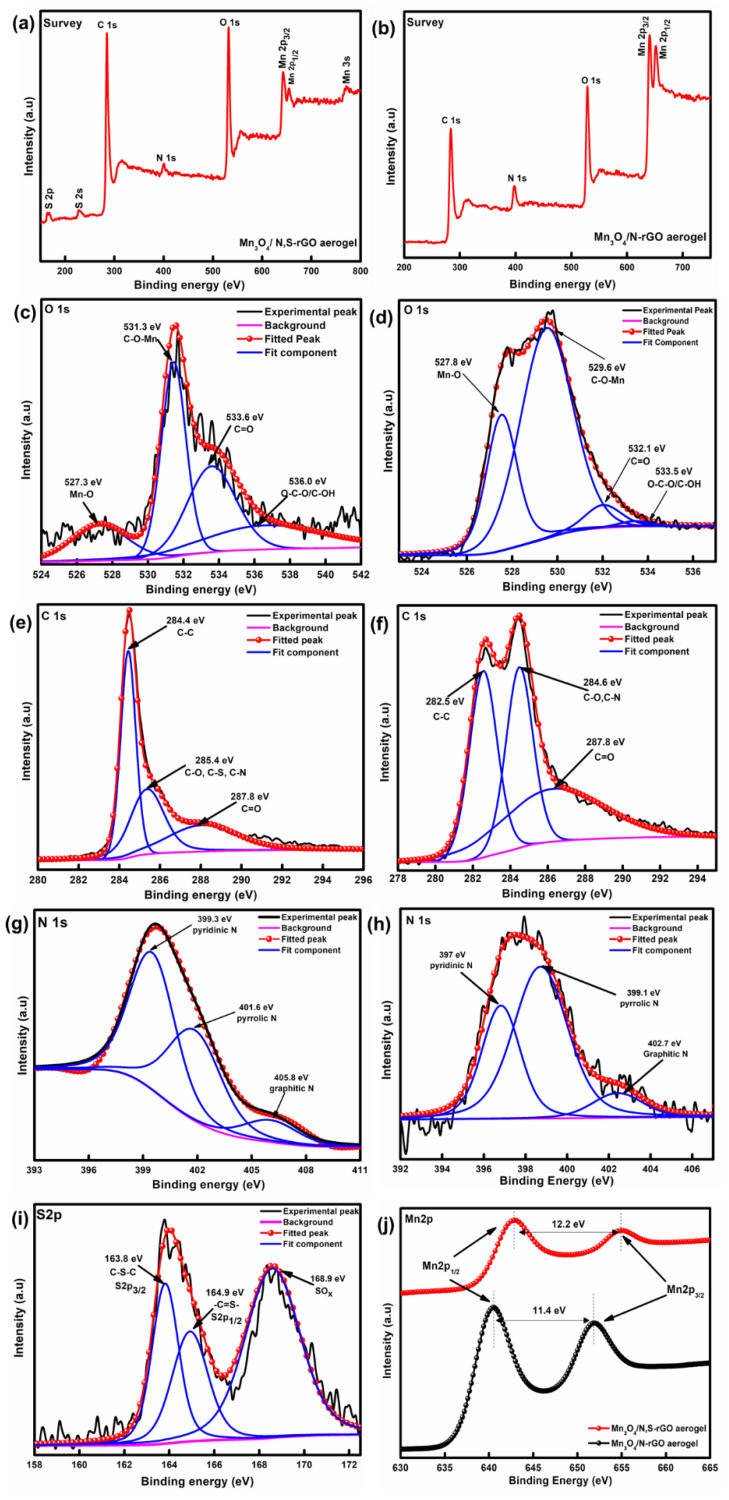
XPS spectra for the survey spectrum of (**a**) Mn_3_O_4_/N,S-rGO aerogel and (**b**) Mn_3_O_4_/N-rGO aerogel, O 1s spectra of (**c**) Mn_3_O_4_/N,S-rGO aerogel and (**d**) Mn_3_O_4_/N-rGO aerogel, C 1s spectra of (**e**) Mn_3_O_4_/N,S-rGO aerogel and (**f**) Mn_3_O_4_/N-rGO aerogel, N 1s spectra of (**g**) Mn_3_O_4_/N,S-rGO aerogel and (**h**) Mn_3_O_4_/N-rGO aerogel, (**i**) S 2p spectra of Mn_3_O_4_/N-rGO aerogel and Mn_3_O_4_/N,S-rGO aerogel and (**j**) deconvoluted spectra of Mn 2p of Mn_3_O_4_/N-rGO aerogel.

The XPS technique was used to obtain further insight into the chemical states of elements on the surface of the samples. Figure 7 presents the XPS spectra of the Mn_3_O_4_/N,S-rGO and Mn_3_O_4_/N-rGO aerogels, respectively. From the survey scan XPS spectra, the presence of nitrogen (Figure 7a) and nitrogen–sulphur (Figure 7b) is noticeable, which agrees well with the FTIR results. For the Mn_3_O_4_/N-rGO aerogel, the atomic percentage of the N was 11.54%, whereas, for the Mn_3_O_4_/N,S-rGO aerogel, the atomic percentages of the N and S were 4.12% and 2.76%, respectively. Both nanocomposites exhibited an Mn 2p_3/2_ at 642 eV, Mn 2p_1/2_ at 653 eV, Mn 3s at 771 eV, an O 1s peak at 531.6 eV, a C 1s peak at 284.5 eV, and N 1s peak at 399.3 eV. For O 1s (Figure 7c,d), the XPS spectra could be deconvoluted into four peaks located at 527, 531, 533, and 534 eV, which correspond to Mn-O, C-O-Mn, C=O, and surface adsorbed oxygen, respectively. The C 1s (Figure 7e,f) could be fitted into three different peaks, which corresponded to the signal of C-C (283.5 eV) and C=O (287 eV) for both nanocomposites, C-O, C-S, and C-N (285.4 eV) for the Mn_3_O_4_/N,S-rGO aerogel, and C-O and C-N (284.6 eV) for the Mn_3_O_4_/N-rGO aerogel. Thus, it further confirmed the presence of N and S-atoms in the nanocomposites [24]. Figure 7g,h present the N 1s region, where the binding energies located at 397, 399, and 403 eV were assigned to pyridinic N, pyrrolic N, and graphitic N, respectively. The presence of pyridinic N, graphitic N, and pyrrolic N at the defect or edge sites was favourable to improving the Na-ion transport and sodium storage capacity [69]. As can be seen from Figure 7i, the S 2p spectra positioned at 163.8, 164.9, and 168.9 eV are assigned to S 2p_3/2_, S p_1/2_, and SO*_x_*, respectively, indicating the existence of Mn-S [70,71]. The curve fitting of the high-resolution Mn 2p spectrum for the Mn_3_O_4_/N-rGO and Mn_3_O_4_/N,S-rGO aerogels is presented in Figure 7j. The peak located at 640–652 eV and 651–655 eV could be assigned to Mn 2p_3/2_ and Mn 2p_1/2_, respectively, indicating the existence of the Mn species, implying the possible presence of Mn_3_O_4_ in the nanocomposites [72]. The splitting widths of Mn 2p_3/2_ and Mn 2p_1/2_ were 12.2 and 11.4 eV for the Mn_3_O_4_/N,S-rGO and Mn_3_O_4_/N-rGO aerogels, respectively, and were in accordance with other earlier reports [73], which further demonstrated that the Mn_3_O_4_ nanoparticles have formed in the Mn_3_O_4_/N-rGO and Mn_3_O_4_/N,S-rGO aerogels.

**Figure 8:**
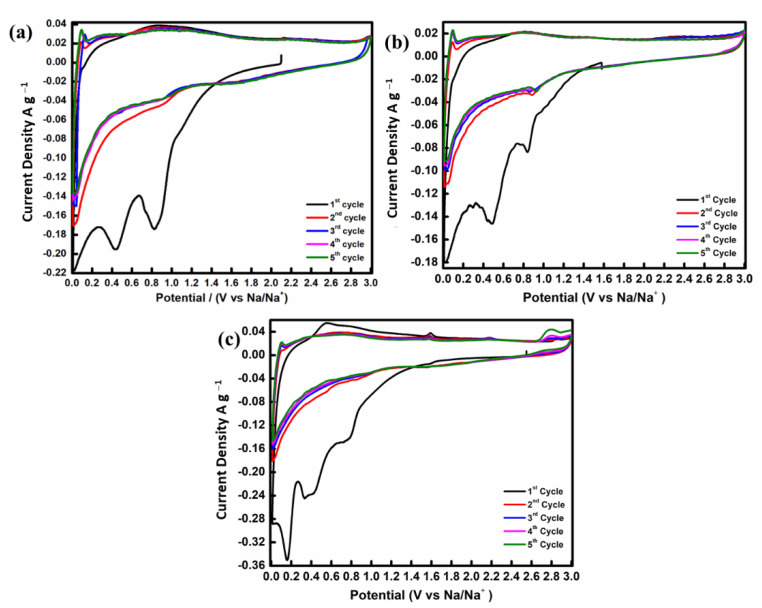
CV curves of (**a**) Mn_3_O_4_ nanoparticles, (**b**) Mn_3_O_4_/N-rGO aerogel, and (**c**) Mn_3_O_4_/N,S-rGO aerogel at a scanning rate of 0.1 mV s^−^^1^.

To evaluate the sodium storage performances of the samples, the CV and galvanostatic charge/discharge testing have been conducted in a half-cell within the potential range of 0.01–3.00 V. As can be seen from Figure 8a, all samples exhibit a strong cathodic peak at 0.85 V in the first cycle and could be ascribed to the reductions of Mn_3_O_4_ to MnO as well as the formation of Na_2_O, which is attributed to the decomposition of the electrolyte according to Equation (1)

Mn_3_O_4_ + 2Na^+^ + 2e^−^ → 3MnO + Na_2_O (1)

The peaks at 0.45 to 0.01 V could be attributed to the reduction of MnO to metallic Mn (Equation (2)) and solid electrolyte interphase (SEI) layer formation on the electrode surface of the electroactive material.

MnO + 2Na^+^ + 2e^−^ → Mn + Na_2_O (2)

During subsequent cycles, the CV curves nearly overlapped, indicating that the Mn_3_O_4_/heteroatom rGO aerogel was reversible during the insertion/de-insertion process of Na^+^ ions [74]. The tiny peaks at 0.1 V and the broad peak at 0.8 V in the anodic process corresponded to Na^+^ extraction into the graphitic carbon layers, which was the inverse process of Na^+^ intercalation. When compared with Li-ion batteries, the CV peaks in Na-ion batteries were broader and weaker. This may be due to the larger radius of Na^+^ than Li^+^ and the slower Na^+^ intercalation between graphitic carbon layers [75,76]. Contrary to the Mn_3_O_4_/N-rGO aerogel (Figure 8b), the Mn_3_O_4_/N,S-rGO aerogel (Figure 8c) exhibited a pair of small redox peaks at 1.6 and 1.8 V, which could be attributed to the sulphur embedded in the porous N,S-rGO aerogel during the Na-ion insertion/de-insertion process [77]. The CV curves exhibited good reversibility, leading to good cycling stability for longer cycles, and the overall sodium storage mechanism between Mn_3_O_4_ and Na^+^ is expressed in Equation (3).

Mn_3_O_4_ + 8Na^+^ + 8e^−^ ↔ 3Mn + 4Na_2_O (3)

**Figure 9:**
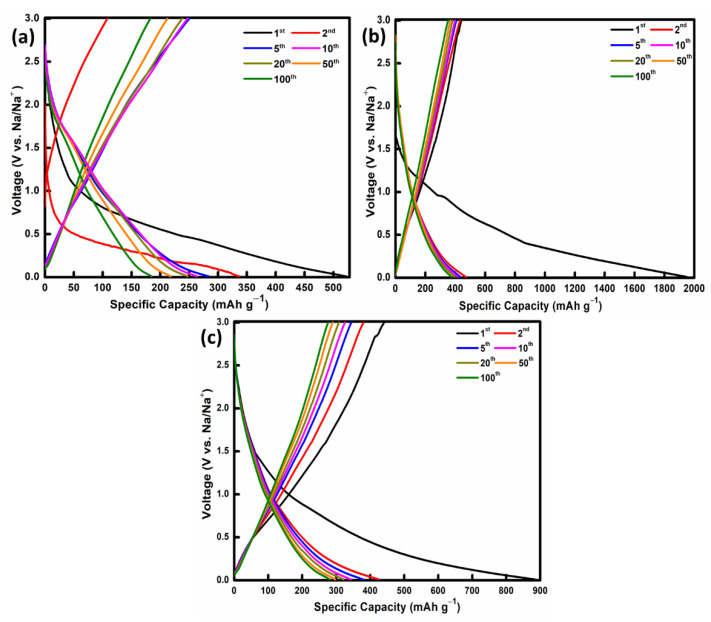
Charge/discharge profiles at selected cycles for (**a**) Mn_3_O_4_ nanoparticles, (**b**) Mn_3_O_4_/N-rGO aerogel, and (**c**) Mn_3_O_4_/N, S-rGO aerogel.

Figure 9 presents the typical discharge/charge profiles of Mn_3_O_4_ and the Mn_3_O_4_/N-rGO and Mn_3_O_4_/N,S-rGO aerogels electrodes for selected cycles at a current density of 0.1 A g^−1^. The subsequent CV curves are different from the first sodiation, and the discharge plateau for the Mn_3_O_4_/N-rGO and Mn_3_O_4_/N,S-rGO aerogels is much longer than the pristine one, indicating that more Na-ions can be inserted into these nanocomposites [78]. Furthermore, no distinct plateau is observed, which is consistent with the CV results.

**Figure 10:**
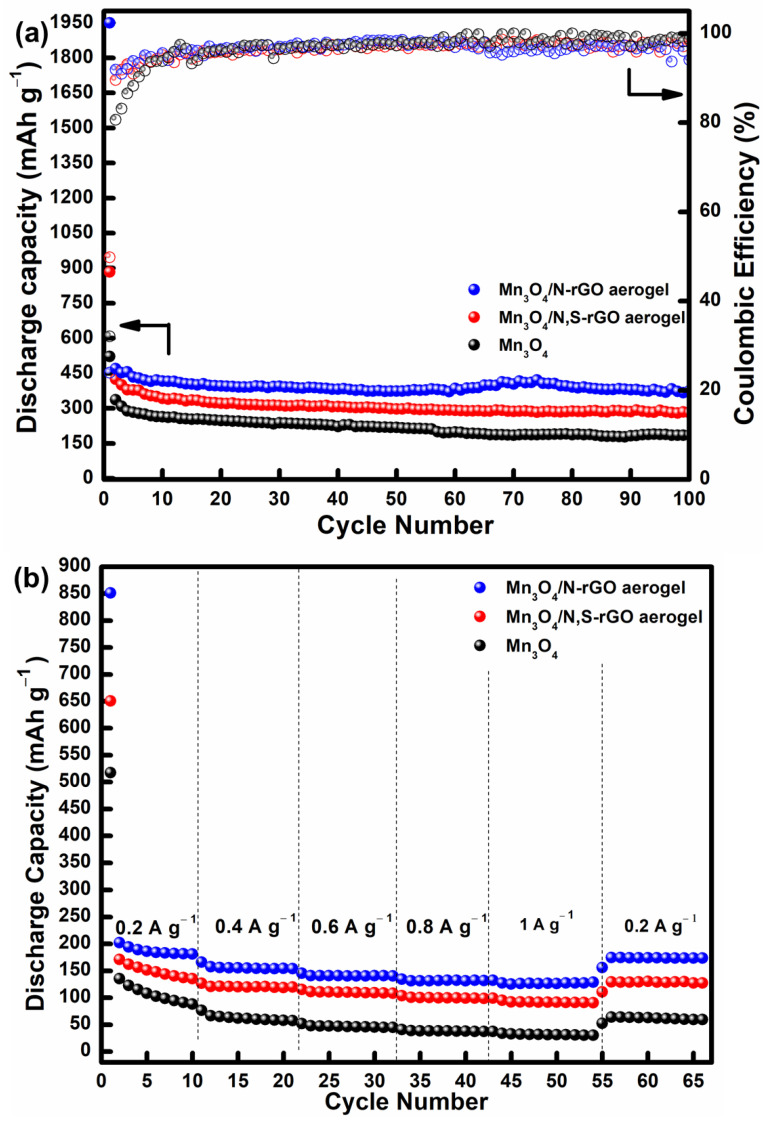
(**a**) Cycling performances and Coulombic efficiencies of up to 100 cycles at a current density of 0.1 A g^−1^ and (**b**) rate capability at different current densities for Mn_3_O_4_, Mn_3_O_4_/N-rGO aerogel, and Mn_3_O_4_/N,S-rGO aerogel.

The cycling stability of all electrodes is presented in Figure 10a. The initial discharge capacities of Mn_3_O_4_, Mn_3_O_4_/N-rGO aerogel and Mn_3_O_4_/N,S-rGO aerogel were measured to be 522, 1950, and 884 mAh g^−1^, respectively. In the second cycle, the discharge capacities were 336, 470, and 425 mAh g^−1^ for Mn_3_O_4_, Mn_3_O_4_/N-rGO aerogel and Mn_3_O_4_/N,S-rGO aerogel, respectively. The irreversible capacity loss was mainly due to the irreversible formation of the SEI layer. Interestingly, the Mn_3_O_4_/N-rGO aerogel maintained its discharge capacity at 374 mAh g^−1^ after 100 cycles with an 80% retention rate. Contrarily, the Mn_3_O_4_/N,S-rGO aerogel and Mn_3_O_4_ exhibited much lower discharge capacities of 281 mAh g^−1^ (68% retention rate) and 185 mAh g^−1^ (55% retention rate) after 100 cycles. For the few initial cycles, the porous structure of rGO aerogel promoted the formation of excessive SEI layers, resulting in a lower initial Coulombic efficiency [79]. Overall, the average Coulombic efficiency of all electrodes was almost 99%. Nevertheless, the Mn_3_O_4_/N,S-rGO aerogel electrodes demonstrated much lower discharge capacity, presumably because of the large atomic radius of S, as well as the large crystallite size than that of Mn_3_O_4_/N-rGO aerogel electrodes.

In addition to their high discharge capacity and good cycling stability, the Mn_3_O_4_/N-rGO and Mn_3_O_4_/N,S-rGO aerogel electrodes exhibited remarkable rate performance, as presented in Figure 10b. The Mn_3_O_4_/N-rGO aerogel electrode exhibited discharge capacities of 203, 166, 145, 135, 128, and 156 mAh g^−1^ at various current densities of 0.2, 0.4, 0.6, 0.8, 1.0, and returning to 0.2 A g^−1^. The minimal drop in capacities with increased current densities indicated a higher degree of reversible Na-ion insertion/de-insertion owing to their expanded interlayer spacing [80]. The improved cycle and rate performance of Mn_3_O_4_/heteroatom-doped rGO aerogels may be due, in part, to the 3D porous structure, which may minimise the electron and ion transport path.

The specific capacity and cyclability of the Mn_3_O_4_/N-rGO aerogel and Mn_3_O_4_/N,S-rGO aerogel were improved, which benefitted from the synergistic effect of the heteroatom doping and porous structure of the rGO, as well as the small particle sizes of Mn_3_O_4_. The rGO sheets as well as the porous structure in the conductive network of the Mn_3_O_4_/heteroatom rGO aerogels provided an efficient electron and ion transport path, thereby decreasing the internal resistance to enhance the reaction kinetics and resulting in a high specific capacity and rate capability [81,82,83]. Poor electrical conductivity and large volume expansion in transition-metal oxide electrodes during the Na-ion insertion/de-insertion processes are among the constraints in the development of these materials for Na-ion battery applications. Here, the rGO sheets are more likely to provide sufficient elastic buffer space for the transition-metal oxide to accommodate the volume expansion/contraction and prevent the electrode from cracking or crumbling during the charge/discharge processes. In addition, the presence of the rGO aerogel could effectively enhance the electrical conductivity of the nanocomposites [84]. Meanwhile, Mn_3_O_4_ nanoparticles anchored on rGO can prevent the restacking of the rGO layers, preserve their high active surface area and maintain the channels for Na-ion diffusion, which is advantageous for increasing the Na storage within the nanocomposites [85]. Doping the rGO aerogels with nitrogen and codoping nitrogen/sulphur improves the physicochemical properties of the rGO component [43]. The incorporation of these heteroatoms into the rGO aerogels could facilitate the charge transfer between adjacent carbon atoms [86,87,88], thus improving the electrical conductivity and electrochemical activity of the rGO itself. The defects created by N-doping and functionalised groups may increase the electrical conductivity, and the larger covalent radius of S compared with Na may increase the interlayer spacing to facilitate Na-ion insertion/de-insertion within the electrode. All the aforementioned characteristics contributed to the improvements in the specific capacity and cycling ability of the Mn_3_O_4_/heteroatom-doped rGO aerogels. This strategy can be used as one of the approaches for mitigating the large volume change and poor electrical conductivity, which is associated with bare transition-metal oxide anodes.

## 4. Conclusions

In summary, the Mn_3_O_4_/heteroatom-doped rGO aerogels have been successfully synthesised via a hydrothermal route, followed by a freeze-drying process using NH_3_ and *L*-cysteine as nitrogen and nitrogen–sulphur sources, respectively. The aerogel structure built well-interconnected heteroatom-doped rGO layers, and the Mn_3_O_4_ nanoparticles distributed on the rGO layers prevented the graphene layers from restacking again. The 3D structure provides a large active surface area and eases electron diffusion and Na-ion transportation. Both the N- and N,S-doped rGO aerogels with Mn_3_O_4_ exhibited high specific capacity, excellent cycling stability, and rate capability than the pristine Mn_3_O_4_. The heteroatom-doped rGO aerogel acts as a robust structure to accommodate the volume expansion of Mn_3_O_4_ nanoparticles and enables reversible Na-ion insertion/de-insertion. Our work demonstrates that N- and N,S-doped rGO aerogels can efficiently improve the Na storage capacity of Mn_3_O_4_ and offer a useful strategy for synthesising high-yield anode materials. Considering the simple step of the preparation process and the excellent cycling stability of the samples, the Mn_3_O_4_/heteroatom-doped rGO aerogel can be considered a potential candidate and provide an opportunity to explore these materials for the next generation of Na-ion batteries.

## Data Availability

Not applicable.

## References

[1] Dunn B., Kamath H., Tarascon J.-M. (2011). Electrical energy storage for the grid: A battery of choices. Science.

[2] Wang H., Chen S., Fu C., Ding Y., Liu G., Cao Y., Chen Z. (2021). Recent advances in conversion-type electrode materials for post lithium-ion batteries. ACS Mater. Lett..

[3] De La Llave E., Borgel V., Park K.-J., Hwang J.-Y., Sun Y.-K., Hartmann P., Chesneau F.-F., Aurbach D. (2016). Comparison between Na-ion and Li-ion cells: Understanding the critical role of the cathodes stability and the anodes pretreatment on the cells behavior. ACS Appl. Mater. Interfaces.

[4] Vaalma C., Buchholz D., Weil M., Passerini S. (2018). A cost and resource analysis of sodium-ion batteries. Nat. Rev. Mater..

[5] Yabuuchi N., Kubota K., Dahbi M., Komaba S. (2014). Research development on sodium-ion batteries. Chem. Rev..

[6] Delmas C. (2018). Sodium and sodium-ion batteries: 50 years of research. Adv. Energy Mater..

[7] Palomares V., Casas-Cabanas M., Castillo-Martínez E., Han M.H., Rojo T. (2013). Update on Na-based battery materials. A growing research path. Energy Environ. Sci..

[8] Rojo T., Hu Y.-S., Forsyth M., Li X. (2018). Sodium-Ion Batteries. Adv. Energy Mater..

[9] Hu Z., Wang L., Zhang K., Wang J., Cheng F., Tao Z., Chen J. (2014). MoS_2_ nanoflowers with expanded interlayers as high-performance anodes for sodium-ion batteries. Angew. Chem. Int. Ed..

[10] Slater M.D., Kim D., Lee E., Johnson C.S. (2013). Correction: Sodium-ion batteries. Adv. Funct. Mater..

[11] Kim H., Kim H., Ding Z., Lee M.H., Lim K., Yoon G., Kang K. (2016). Recent progress in electrode materials for sodium-ion batteries. Adv. Energy Mater..

[12] Larcher D., Tarascon J.-M. (2015). Towards greener and more sustainable batteries for electrical energy storage. Nat. Chem..

[13] Li Y., Lu Y., Zhao C., Hu Y.-S., Titirici M.-M., Li H., Huang X., Chen L. (2017). Recent advances of electrode materials for low-cost sodium-ion batteries towards practical application for grid energy storage. Energy Storage Mater..

[14] Jamesh M.I., Prakash A.S. (2018). Advancement of technology towards developing Na-ion batteries. J. Power Sources.

[15] Gu M., Kushima A., Shao Y., Zhang J.-G., Liu J., Browning N.D., Li J., Wang C. (2013). Probing the failure mechanism of SnO_2_ nanowires for sodium-ion batteries. Nano Lett..

[16] Gao H., Ji B., Jäger I.L., Arzt E., Fratzl P. (2003). Materials become insensitive to flaws at nanoscale: Lessons from nature. Proc. Natl. Acad. Sci. USA.

[17] Yusoff N.F.M., Idris N.H., Din M.F.M., Majid S.R., Harun N.A., Rahman M. (2020). Investigation on the electrochemical performances of Mn_2_O_3_ as a potential anode for Na-ion batteries. Sci. Rep..

[18] Li H., Liu A., Zhao S., Guo Z., Wang N., Ma T. (2018). In situ growth of a feather-like MnO_2_ nanostructure on carbon paper for high-performance rechargeable sodium-ion batteries. ChemElectroChem.

[19] Li T., Qin A., Yang L., Chen J., Wang Q., Zhang D., Yang H. (2017). In situ grown Fe_2_O_3_ single crystallites on reduced graphene oxide nanosheets as high performance conversion anode for sodium-ion batteries. ACS Appl. Mater. Interfaces.

[20] Fu Y., Wei Q., Wang X., Zhang G., Shu H., Yang X., Tavares A.C., Sun S. (2016). A facile synthesis of Fe_3_O_4_ nanoparticles/graphene for high-performance lithium/sodium-ion batteries. RSC Adv..

[21] Xu M., Xia Q., Yue J., Zhu X., Guo Q., Zhu J., Xia H. (2019). Rambutan-like hybrid hollow spheres of carbon confined Co_3_O_4_ nanoparticles as advanced anode materials for sodium-ion batteries. Adv. Funct. Mater..

[22] Liu Y., Yang Y., Wang X., Dong Y., Tang Y., Yu Z., Zhao Z., Qiu J. (2017). Flexible paper-like free-standing electrodes by anchoring ultrafine SnS_2_ nanocrystals on graphene nanoribbons for high-performance sodium ion batteries. ACS Appl. Mater. Interfaces.

[23] Ji L., Gu M., Shao Y., Li X., Engelhard M.H., Arey B.W., Wang W., Nie Z., Xiao J., Wang C. (2014). Controlling SEI formation on SnSb-porous carbon nanofibers for improved Na ion storage. Adv. Mater..

[24] Qin Y., Jiang Z., Guo L., Huang J., Jiang Z.-J. (2021). Controlled thermal oxidation derived Mn_3_O_4_ encapsulated in nitrogen doped carbon as an anode for lithium/sodium ion batteries with enhanced performance. Chem. Eng. J..

[25] Wang B., Li F., Wang X., Wang G., Wang H., Bai J. (2019). Mn_3_O_4_ nanotubes encapsulated by porous graphene sheets with enhanced electrochemical properties for lithium/sodium-ion batteries. Chem. Eng. J..

[26] Deng Y., Wan L., Xie Y., Qin X., Chen G. (2014). Recent advances in Mn-based oxides as anode materials for lithium ion batteries. RSC Adv..

[27] Duan J., Zheng Y., Chen S., Tang Y., Jaroniec M., Qiao S. (2016). Correction: Mesoporous hybrid material composed of Mn_3_O_4_ nanoparticles on nitrogen-doped graphene for highly efficient oxygen reduction reaction. Chem. Commun..

[28] Jiang Y., Hu M., Zhang D., Yuan T., Sun W., Xu B., Yan M. (2014). Transition metal oxides for high performance sodium ion battery anodes. Nano Energy.

[29] Hao Q., Liu B., Ye J., Xu C. (2017). Well encapsulated Mn_3_O_4_ octahedra in graphene nanosheets with much enhanced Li-storage performances. J. Colloid Interface Sci..

[30] Wu Y., Liu S., Wang H., Wang X., Zhang X., Jin G. (2013). A novel solvothermal synthesis of Mn_3_O_4_/graphene composites for supercapacitors. Electrochim. Acta.

[31] Zhang T., Zhu C., Shi Y., Li Y., Zhu S., Zhang D. (2017). Synthesis of Fe_2_O_3_ in situ on the surface of mesoporous carbon from alginate as a high-performance anode for lithium-ion batteries. Mater. Lett..

[32] Li Y., Zhao Y., Ma C., Shi J., Zhao Y. (2020). Highly monodispersed graphene/SnO_2_ hybrid nanosheets as bifunctional anode materials of Li-ion and Na-ion batteries. J. Alloys Compd..

[33] Wang H., Cui L.F., Yang Y., Sanchez Casalongue H., Robinson J.T., Liang Y., Cui Y., Dai H. (2010). Mn_3_O_4_-graphene hybrid as a high-capacity anode material for lithium ion batteries. J. Am. Chem. Soc..

[34] Long J., Yang Z., Yang F., Cuan J., Wu J. (2020). Electrospun core-shell Mn_3_O_4_/carbon fibers as high-performance cathode materials for aqueous zinc-ion batteries. Electrochim. Acta.

[35] Mao J., Zhou T., Zheng Y., Gao H., kun Liu H., Guo Z. (2018). Two-dimensional nanostructures for sodium-ion battery anodes. J. Mater. Chem. A.

[36] Li Y., Ye D., Liu W., Shi B., Guo R., Pei H., Xie J. (2017). A three-dimensional core-shell nanostructured composite of polypyrrole wrapped MnO_2_/reduced graphene oxide/carbon nanotube for high performance lithium ion batteries. J. Colloid Interface Sci..

[37] Zhao D., Wang L., Yu P., Zhao L., Tian C., Zhou W., Zhang L., Fu H. (2015). From graphite to porous graphene-like nanosheets for high rate lithium-ion batteries. Nano Res..

[38] Zhou C., Zhang K., Hong M., Yang Y., Hu N., Su Y., Zhang L., Zhang Y. (2020). Laser-induced MnO/Mn_3_O_4_/N-doped-graphene hybrid as binder-free anodes for lithium ion batteries. Chem. Eng. J..

[39] Guo C., Xie Y., Pan K., Li L. (2020). MOF-derived hollow SiO_x_ nanoparticles wrapped in 3D porous nitrogen-doped graphene aerogel and their superior performance as the anode for lithium-ion batteries. Nanoscale.

[40] Vrettos K., Angelopoulou P., Papavasiliou J., Avgouropoulos G., Georgakilas V. (2020). Sulfur-doped graphene aerogels reinforced with carbon fibers as electrode materials. J. Mater. Sci..

[41] Yao Z., Xia X., Xie D., Wang Y., Zhou C.-A., Liu S., Deng S., Wang X., Tu J. (2018). Enhancing ultrafast lithium ion storage of Li_4_Ti_5_O_12_ by tailored TiC/C core/shell skeleton plus nitrogen doping. Adv. Funct. Mater..

[42] Xiong Q.Q., Lou J.J., Teng X.J., Lu X.X., Liu S.Y., Chi H.Z., Ji Z.G. (2018). Controllable synthesis of N-C@LiFePO_4_ nanospheres as advanced cathode of lithium ion batteries. J. Alloys Compd..

[43] Jing M., Chen Z., Li Z., Li F., Chen M., Zhou M., He B., Chen L., Hou Z., Chen X. (2018). Facile synthesis of ZnS/N,S co-doped carbon composite from zinc metal complex for high-performance sodium-ion batteries. ACS Appl. Mater. Interfaces.

[44] Wang Z., Qie L., Yuan L., Zhang W., Hu X., Huang Y. (2013). Functionalized N-doped interconnected carbon nanofibers as an anode material for sodium-ion storage with excellent performance. Carbon.

[45] Wang X., Li G., Hassan F.M., Li J., Fan X., Batmaz R., Xiao X., Chen Z. (2015). Sulfur covalently bonded graphene with large capacity and high rate for high-performance sodium-ion batteries anodes. Nano Energy.

[46] Liu Y., Qiao Y., Wei G., Li S., Lu Z., Wang X., Lou X. (2018). Sodium storage mechanism of N, S co-doped nanoporous carbon: Experimental design and theoretical evaluation. Energy Storage Mater..

[47] Kumar R., Sahoo S., Joanni E., Singh R.K., Maegawa K., Tan W.K., Kawamura G., Kar K.K., Matsuda A. (2020). Heteroatom doped graphene engineering for energy storage and conversion. Mater. Today.

[48] Hummers W.S., Offeman R.E. (1958). Preparation of graphitic oxide. J. Am. Chem. Soc..

[49] Mahamad Yusoff N.F., Idris N.H., Md Din M.F., Majid S.R., Harun N.A., Rahman M.M. (2020). Electrochemical sodiation/desodiation into Mn_3_O_4_ nanoparticles. ACS Omega.

[50] Chen J., Yao B., Li C., Shi G. (2013). An improved Hummers method for eco-friendly synthesis of graphene oxide. Carbon.

[51] Zhang L., Shi G. (2011). Preparation of highly conductive graphene hydrogels for fabricating supercapacitors with high rate capability. J. Phys. Chem. C.

[52] Wang P., Zhang Y., Yin Y., Fan L., Zhang N., Sun K. (2018). In situ synthesis of CuCo_2_S_4_@ N/S-doped graphene composites with pseudocapacitive properties for high-performance lithium-ion batteries. ACS Appl. Mater. Interfaces.

[53] Elsiddig Z.A., Wang D., Xu H., Zhang W., Zhang T., Zhang P., Tian W., Sun Z., Chen J. (2018). Three-dimensional nitrogen-doped graphene wrapped LaMnO_3_ nanocomposites as high-performance supercapacitor electrodes. J. Alloys Compd..

[54] Ma X., Zhang P., Zhao Y., Liu Y., Li J., Zhou J.Y., Pan X., Xie E. (2017). Role of N doping on the electrochemical performances of ZnCo_2_O_4_ quantum dots/reduced graphene oxide composite nanosheets. Chem. Eng. J..

[55] Kang J.-H., Chen J.-S. (2018). Using ethylenediamine to prepare three dimensional nitrogen-doped graphene aerogel/sulfur composite for lithium-sulfur batteries. Diam. Relat. Mater..

[56] Shi P., Su R., Wan F., Zhu M., Li D., Xu S. (2012). Co_3_O_4_ nanocrystals on graphene oxide as a synergistic catalyst for degradation of Orange II in water by advanced oxidation technology based on sulfate radicals. Appl. Catal. B.

[57] Liang Y., Li Y., Wang H., Zhou J., Wang J., Regier T., Dai H. (2011). Co_3_O_4_ nanocrystals on graphene as a synergistic catalyst for oxygen reduction reaction. Nat. Mater..

[58] Cheng Y., Li B., Wei Z., Wang Y., Wei D., Jia D., Feng Y., Zhou Y. (2020). Mn_3_O_4_ tetragonal bipyramid laden nitrogen doped and hierarchically porous carbon composite as positive electrode for high-performance asymmetric supercapacitor. J. Power Sources.

[59] Kim H., Kim S.-W., Hong J., Park Y.-U., Kang K. (2011). Electrochemical and ex-situ analysis on manganese oxide/graphene hybrid anode for lithium rechargeable batteries. J. Mater. Res..

[60] Zhang J., Li C., Peng Z., Liu Y., Zhang J., Liu Z., Li D. (2017). 3D free-standing nitrogen-doped reduced graphene oxide aerogel as anode material for sodium ion batteries with enhanced sodium storage. Sci. Rep..

[61] Xia Y., Xiao Z., Dou X., Huang H., Lu X., Yan R., Gan Y., Zhu W., Tu J., Zhang W. (2013). Green and facile fabrication of hollow porous MnO/C microspheres from microalgaes for lithium-ion batteries. ACS Nano.

[62] Bernard M.C., Hugot-Le Goff A., Thi B.V., de Torresi S.C. (1993). Electrochromic reactions in manganese oxides: I. Raman analysis. J. Electrochem. Soc..

[63] Julien C., Massot M., Poinsignon C. (2004). Lattice vibrations of manganese oxides: Part I. Periodic structures. Spectrochim. Acta Part A.

[64] Wang Y., Zhu L., Yang X., Shao E., Deng X., Liu N., Wu M. (2015). Facile synthesis of three-dimensional Mn_3_O_4_ hierarchical microstructures and their application in the degradation of methylene blue. J. Mater. Chem. A.

[65] Wang J.-G., Jin D., Zhou R., Li X., Liu X.-R., Shen C., Xie K., Li B., Kang F., Wei B. (2016). Highly flexible graphene/Mn_3_O_4_ nanocomposite membrane as advanced anodes for Li-ion batteries. ACS Nano.

[66] Ouyang Z., Lei Y., Chen Y., Zhang Z., Jiang Z., Hu J., Lin Y. (2019). Preparation and specific capacitance properties of sulfur, nitrogen co-doped graphene quantum dots. Nanoscale Res. Lett..

[67] Meng J., Cao Y., Suo Y., Liu Y., Zhang J., Zheng X. (2015). Facile fabrication of 3D SiO_2_@graphene aerogel composites as anode material for lithium ion batteries. Electrochim. Acta.

[68] Rochman R.A., Wahyuningsih S., Ramelan A.H., Hanif Q.A. (2019). Preparation of nitrogen and sulphur Co-doped reduced graphene oxide (rGO-NS) using N and S heteroatom of thiourea. IOP Conf. Ser. Mater. Sci. Eng..

[69] Meng S., Zhao D.-L., Wu L.-L., Ding Z.-W., Cheng X.-W., Hu T. (2018). Fe_2_O_3_/nitrogen-doped graphene nanosheet nanocomposites as anode materials for sodium-ion batteries with enhanced electrochemical performance. J. Alloys Compd..

[70] Rani K.K., Karuppiah C., Wang S.-F., Alaswad S.O., Sireesha P., Devasenathipathy R., Jose R., Yang C.-C. (2020). Direct pyrolysis and ultrasound assisted preparation of N, S co-doped graphene/Fe3C nanocomposite as an efficient electrocatalyst for oxygen reduction and oxygen evolution reactions. Ultrason. Sonochem..

[71] Jiang T., Yin N., Bai Z., Dai P., Yu X., Wu M., Li G. (2018). Wet chemical synthesis of S doped Co_3_O_4_ nanosheets/reduced graphene oxide and their application in dye sensitized solar cells. Appl. Surf. Sci..

[72] Tan B.J., Klabunde K.J., Sherwood P.M. (1991). XPS studies of solvated metal atom dispersed (SMAD) catalysts. Evidence for layered cobalt-manganese particles on alumina and silica. J. Am. Chem. Soc..

[73] Yang S., Liu L., Wang G., Li G., Deng D., Qu L. (2015). One-pot synthesis of Mn_3_O_4_ nanoparticles decorated with nitrogen-doped reduced graphene oxide for sensitive nonenzymatic glucose sensing. J. Electroanal. Chem..

[74] He Y., Xu P., Zhang B., Du Y., Song B., Han X., Peng H. (2017). Ultrasmall MnO nanoparticles supported on nitrogen-doped carbon nanotubes as efficient anode materials for sodium ion batteries. ACS Appl. Mater. Interfaces.

[75] Valvo M., Philippe B., Lindgren F., Tai C.-W., Edström K. (2016). Insight into the processes controlling the electrochemical reactions of nanostructured iron oxide electrodes in Li-and Na-half cells. Electrochim. Acta.

[76] Wang X., Liu X., Wang G., Xia Y., Wang H. (2016). One-dimensional hybrid nanocomposite of high-density monodispersed Fe_3_O_4_ nanoparticles and carbon nanotubes for high-capacity storage of lithium and sodium. J. Mater. Chem. A.

[77] Zhang Y., Zhao Y., Bakenov Z., Tuiyebayeva M., Konarov A., Chen P. (2014). Synthesis of hierarchical porous sulfur/polypyrrole/multiwalled carbon nanotube composite cathode for lithium batteries. Electrochim. Acta.

[78] Lin Z., Xiong X., Zheng J., Wang G., Yang C. (2017). Three-dimensional N-doped graphene as anode material with superior cycle stability for sodium ion batteries. Mater. Lett..

[79] Ye J., Zhao H., Song W., Wang N., Kang M., Li Z. (2019). Enhanced electronic conductivity and sodium-ion adsorption in N/S co-doped ordered mesoporous carbon for high-performance sodium-ion battery anode. J. Power Sources.

[80] Sahu T.S., Mitra S. (2015). Exfoliated MoS_2_ sheets and reduced graphene oxide-an excellent and fast anode for sodium-ion battery. Sci. Rep..

[81] Li D., Yan D., Ma J., Qin W., Zhang X., Lu T., Pan L. (2016). One-step microwave-assisted synthesis of Sb_2_O_3_/reduced graphene oxide composites as advanced anode materials for sodium-ion batteries. Ceram. Int..

[82] Zhang X., Chen T., Yan D., Qin W., Hu B., Sun Z., Pan L. (2015). MgFe_2_O_4_/reduced graphene oxide composites as high-performance anode materials for sodium ion batteries. Electrochim. Acta.

[83] Zhang Z.-J., Wang Y.-X., Chou S.-L., Li H.-J., Liu H.-K., Wang J.-Z. (2015). Rapid synthesis of α-Fe_2_O_3_/rGO nanocomposites by microwave autoclave as superior anodes for sodium-ion batteries. J. Power Sources.

[84] Sun J., Lee H.-W., Pasta M., Yuan H., Zheng G., Sun Y., Li Y., Cui Y. (2015). A phosphorene–graphene hybrid material as a high-capacity anode for sodium-ion batteries. Nat. Nanotechnol..

[85] Liu X., Chen T., Chu H., Niu L., Sun Z., Pan L., Sun C.Q. (2015). Fe_2_O_3_-reduced graphene oxide composites synthesized via microwave-assisted method for sodium ion batteries. Electrochim. Acta.

[86] Wu Z.-S., Ren W., Xu L., Li F., Cheng H.-M. (2011). Doped graphene sheets as anode materials with superhigh rate and large capacity for lithium ion batteries. ACS Nano.

[87] Jeong H.M., Lee J.W., Shin W.H., Choi Y.J., Shin H.J., Kang J.K., Choi J.W. (2011). Nitrogen-doped graphene for high-performance ultracapacitors and the importance of nitrogen-doped sites at basal planes. Nano Lett..

[88] Wu Z.-S., Winter A., Chen L., Sun Y., Turchanin A., Feng X., Müllen K. (2012). Three-dimensional nitrogen and boron co-doped graphene for high-performance all-solid-state supercapacitors. Adv. Mater..

